# Effect of PAIP1 on the metastatic potential and prognostic significance in oral squamous cell carcinoma

**DOI:** 10.1038/s41368-022-00162-8

**Published:** 2022-02-14

**Authors:** Neeti Swarup, Kyoung-Ok Hong, Kunal Chawla, Su-Jung Choi, Ji-Ae Shin, Kyu-Young Oh, Hye-Jung Yoon, Jae-Il Lee, Sung-Dae Cho, Seong-Doo Hong

**Affiliations:** 1 Department of Oral Pathology, School of Dentistry and Dental Research Institute, Seoul National University, Seoul, Republic of Korea; 2 Department of Computer Science, School of Interactive Computing, Georgia Institute of Technology, Atlanta, GA USA

**Keywords:** Cancer, Oral cancer, Genetic databases, Oral cancer detection

## Abstract

Poly Adenylate Binding Protein Interacting protein 1 (PAIP1) plays a critical role in translation initiation and is associated with the several cancer types. However, its function and clinical significance have not yet been described in oral squamous cell carcinoma (OSCC) and its associated features like lymph node metastasis (LNM). Here, we used the data available from Gene Expression Omnibus (GEO), The Cancer Genome Atlas (TCGA), and Clinical Proteomic Tumor Analysis Consortium (CPTAC) to analyze PAIP1 expression in oral cancer. The publicly available data suggests that PAIP1 mRNA and protein levels were increased in OSCC. The high PAIP1 expression was more evident in samples with advanced stage, LNM, and worse pattern of invasion. Moreover, the in vitro experiments revealed that PAIP1 knockdown attenuated colony forming, the aggressiveness of OSCC cell lines, decreasing MMP9 activity and SRC phosphorylation. Importantly, we found a correlation between PAIP1 and pSRC through the analysis of the IHC scores and CPTAC data in patient samples. Our findings suggest that PAIP1 could be an independent prognostic factor in OSCC with LNM and a suitable therapeutic target to improve OSCC patient outcomes.

## Introduction

Tumor metastasis is critical for tumor progression, depending on motility and invasion, cancer cell plasticity, the modulation of the microenvironment, and cancer cell colonization^1^. Like most epithelial cancers, OSCC is a common cancer type of the mucosal linings of the oral cavity. LNM is one of the deterministic prognostic indicators in OSCC^2^. Despite the multimodal approach, including surgery or radiotherapy as treatment approaches in patients with OSCC, the low LNM-associated survival rate of less than 50% remains a major concern^3,4^. Thus, there is crucial to identify novel LNM-related biomarkers to improve the clinical outcomes in patients with OSCC.

The coding gene of PAIP1is located at 5p12 chromosomal location, encoding a protein of 479 amino acids sharing homology with the central domain of human eukaryotic initiation factor 4G (eIF4G), consequently interacting with the eIF4A and eIF3 complexes to facilitate translation initiation^5–9^. PAIP1 displays a PAM1 and PAM2 domain, of which PAM1 interacts with the RRM 1and 2 domains of the Poly Adenylate Binding Protein (PABP) and PAM2 with the C-terminal domain of PABP^5^. Its interaction with eIF3 is regulated via the S6 kinase^10^. These interactions help the circularization of the RNA and translation initiation^8^.

Since its identification by Sonenberg et al. in 1998, it has been described to be frequently upregulated in several types of cancer. In breast cancer, the high PAIP1 expression was related to late clinical stage, high histologic grade, and a low survival rate^11^. Similarly, it reportedly regulates different aspects of oncogenesis, such as cellular proliferation, cell death, and metastasis in several cancers^12–15^. Although these findings suggest that PAIP1 plays a part in tumor progression and metastasis as a potential prognostic factor, the correlation between PAIP1 expression and OSCC metastasis has not yet been described.

In this study, we tried to examine whether PAIP1 is frequently overexpressed in human OSCC tissues and cell lines and to investigate the correlation between PAIP1 expression and the clinicopathological parameters of patients with OSCC, just as well as to evaluate the prognostic value of PAIP. Furthermore, to explore the function of PAIP1 and its underlying molecular mechanisms, we observed the migration and invasion in PAIP1-silenced OSCC cell lines.

## Results

### PAIP1 was upregulated in OSCCs

**Fig. 1:**
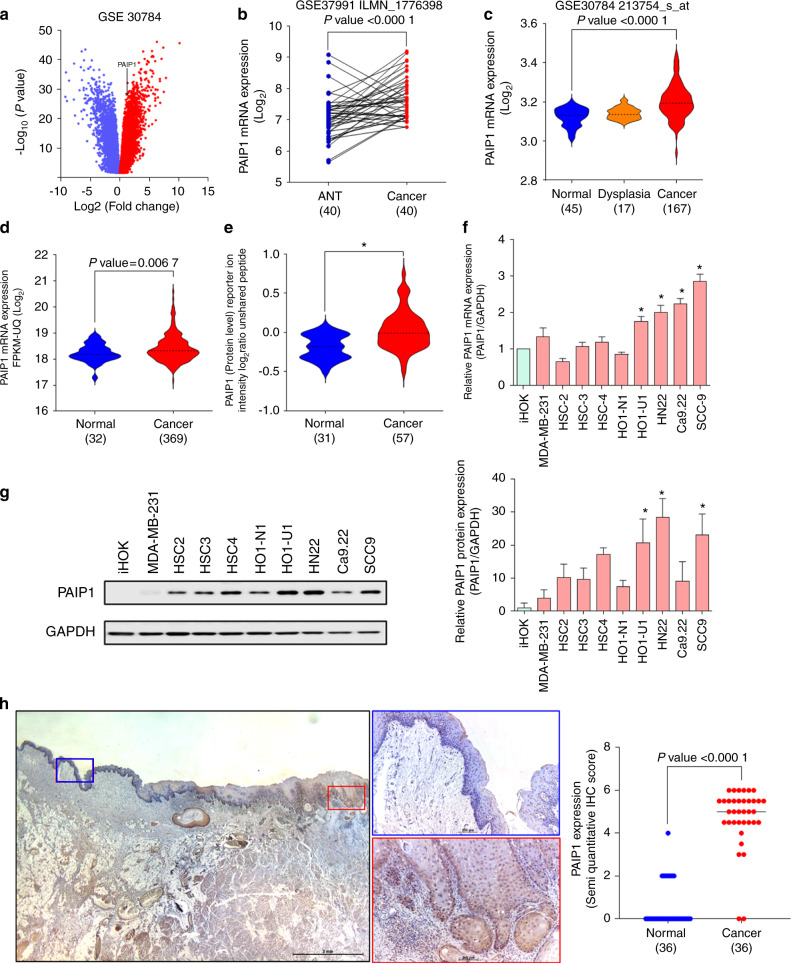
PAIP1 is frequently upregulated in OSCC. **a** Volcano plot depicting significantly differentially expressed genes, in 45 normal and 45 cancer samples from GSE30784, upregulated and downregulated genes were represented red and blue, respectively. PAIP1 log2(fold change) 1.217, −log10 (*P*-value) 28.329. **b**, **c** PAIP1 mRNA levels were significantly upregulated in custom OSCC patient cohorts, compared with paired and unpaired normal tissue. Results were retrieved from GSE37991 and 30784, GEO database, respectively. In **b**, each line indicated one pair of matched adjacent normal-tumor samples. **d**, **e** PAIP1 mRNA and protein levels in oral cancer when compared to normal, and the results were retrieved from TCGA and CPTAC, respectively. **f**, **g** PAIP1 mRNA and protein were expressed across various OSCC cells and MDA-MB-231 cells, compared with iHOK cells. **h** Images and plots indicate the PAIP1 levels in OSCC patients. The colored boxes indicate cropped fields of adjacent normal mucosa (blue) and tumor (red) tissues. The dashed line represents the median. *P < 0.05, as evaluated using a student t-test or one-way ANOVA.

To scrutinize the genetic alteration and expression of PAIP1 in various cancers, we first performed *in silico* PAIP1 expression analyses. In public database, PAIP1 was frequently amplified and overexpressed in a variety of cancers including head and neck squamous cell carcinoma (HNSCC) (Fig. S1a, b). We also found that the copy number was significantly associated with the mRNA levels in the TCGA and CCLE database for HNSCC (Fig. S1c, d, with *R*-value of 0.81 and 0.707, respectively). To further evaluate the PAIP1 expression in HNSCC, we assessed differentially expressed genes in 45 healthy and 45 cancer samples from the GSE dataset 30784 and found different, significantly upregulated PAIP1 transcripts (Fig. 1a). Compared to the paired normal adjacent mucosa, the PAIP1 mRNA levels significantly increased in the HNSCC tissues and were upregulated during the carcinogenetic process (Fig. 1b–d). Moreover, the upregulated PAIP1 protein levels appeared to correlate with the HNSCC patient cohorts from the CPTAC database (Fig. 1e). Consistent with *in silico* analyses, the PAIP1 mRNA and protein levels were frequently high in various OSCC cell lines (Fig. 1f, g). Based on semi-quantitative IHC scores, the PAIP1 levels significantly enhanced in the OSCC tissue, compared with the healthy oral epithelium (Fig. 1h). These results suggest that the PAIP1 amplification and overexpression correlated with OSCC progression.

### The PAIP1 expression significantly correlated with OSCC prognostic implications

**Fig. 2:**
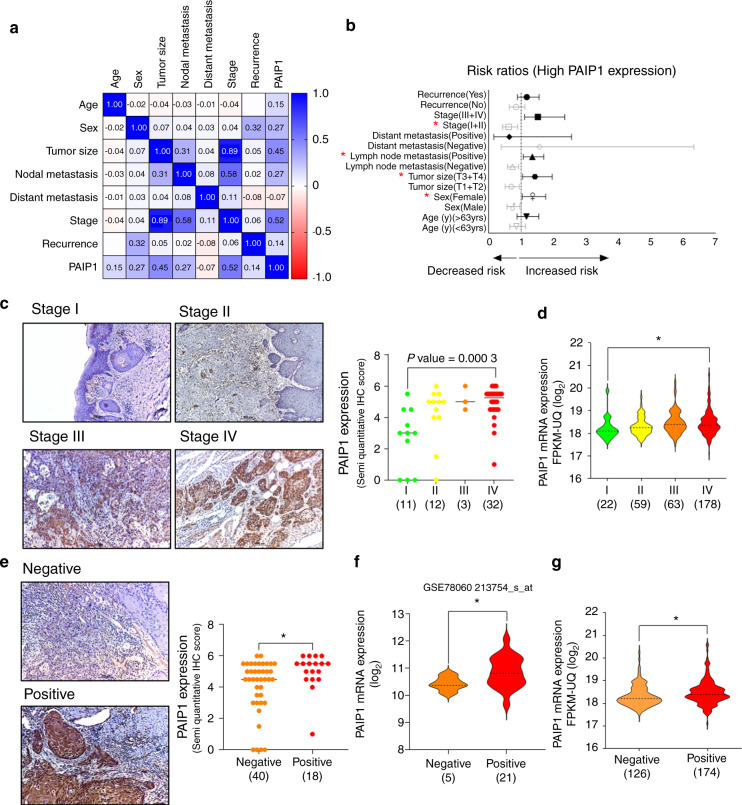
PAIP1 expression is associated with clinicopathologic features in OSCC. **a** Heat map demonstrating the correlation between PAIP1 and different clinicopathological variables. **b** Forest plot represents relative risks associated with high PAIP1 expression. Variables with *P* values < 0.05 are marked with *(red). **c** Images and plots indicate the protein levels of PAIP1 across different stages of patients with OSCC. **d** Violin plots show PAIP1 mRNA levels across different stages of HNSCC patient cohorts from TCGA datasets. **e** Images and plots indicate the protein levels of PAIP1 in patients with OSCC and LNM. **f**, **g** Violin plots indicate PAIP1 mRNA levels in custom OSCC patient cohorts with LNM from GSE78060 and TCGA datasets, respectively. The dashed line represents the median. *P < 0.05, as evaluated using a student t-test or one-way ANOVA.

To further investigate the correlation between PAIP1 and OSCC progression, we performed a multivariable analysis between the clinicopathologic variables and PAIP1 expression. We found that high PAIP1 expressions significantly correlated with gender, tumor size, LNM, and stage, exhibiting a high-risk ratio (Fig. 2a, b and Supplementary Table 3). Figure 2c shows that the upregulation of the PAIP1 levels appeared to correlate with worsening TMN stages of patients with OSCC, similar to *in silico* data (Fig. 2d). Moreover, the PAIP1 expression significantly increased in cases of nodal metastasis. This observation was in accord with the results of the GSE78060 and TCGA custom cohorts (Fig. 2e–g). Notably, a close relationship could be observed between the PAIP1 expression and the survival of OSCC patients that high expression of PAIP1 was associated with poor overall survival of OSCC patients observed using KM plotter (Fig. S3). These results data that a high PAIP1 level might be a risk factor indicating a worse OSCC prognosis.

### The PAIP1 expression pattern was distinguished in different tumor regions with histopathological features

**Fig. 3:**
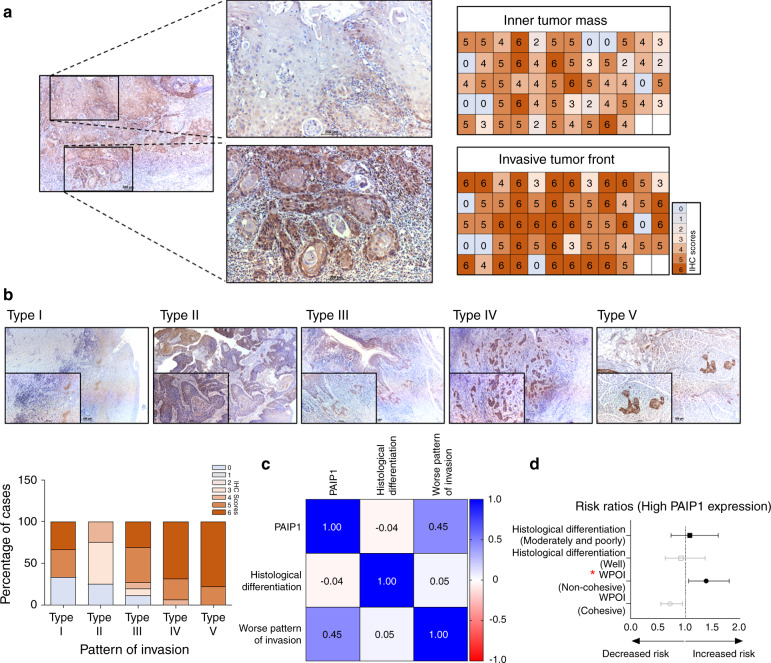
PAIP1 expression is associated with histopathologic features in OSCC. **a** Images and heat maps represent the expression patterns of PAIP1 protein in ITM and ITF of OSCC. **b** Representative images and graphs show PAIP1 expression levels in different types of POI. The red line in the Type V image indicates 1 mm of normal tissue between the main tumor and tumor islands. **c** Heat map demonstrating the correlation between PAIP1 and different histopathological variables. **d** Forest plot represents relative risks of different histopathological variables associated with high PAIP1 expression. Variables with *P* < 0.05 are marked with *(red).

To determine whether PAIP1 has a reliable predictive value for OSCC prognosis, we assessed its association with the histopathological parameters in different regions classified as ITM (inner tumor mass) and ITF (invasive tumor front). The IHC scores of the PAIP1 staining in ITM or ITF are shown as a heat map. The ITF exhibited significantly higher IHC scores than ITM (Fig. 3a and Fig. S4a, *P* < 0.000 1). In a recent comparative study, the worst pattern of invasion (WPOI) was reportedly superior to histological grade as a prognostic indicator of OSCC^16^. To investigate the relevance of PAIP1 expression on WPOI, we categorized it into 5 different patterns and classified it into two categories: cohesive and non-cohesive, following their grading schemes (Supplementary Tables 2 and 4). As illustrated in Fig. 3b, Type IV and V showed high PAIP1 expression, compared to Type I–III. In addition, PAIP1 significantly correlated more with WPOI than the histological differentiation (Fig. 3c). Using multivariable analysis, we found that increased PAIP1 expression was related to increased risk of WPOI (non-cohesive patterns of invasion) with a relative risk of 1.38 (95% CI 1.06, 1.8) (Fig. 3d and Supplementary Table 4). These results suggest that PAIP1 contributes to OSCC invasiveness by varying the level of expression.

### The PAIP1 knockdown attenuated OSCC cell migration and invasion

**Fig. 4:**
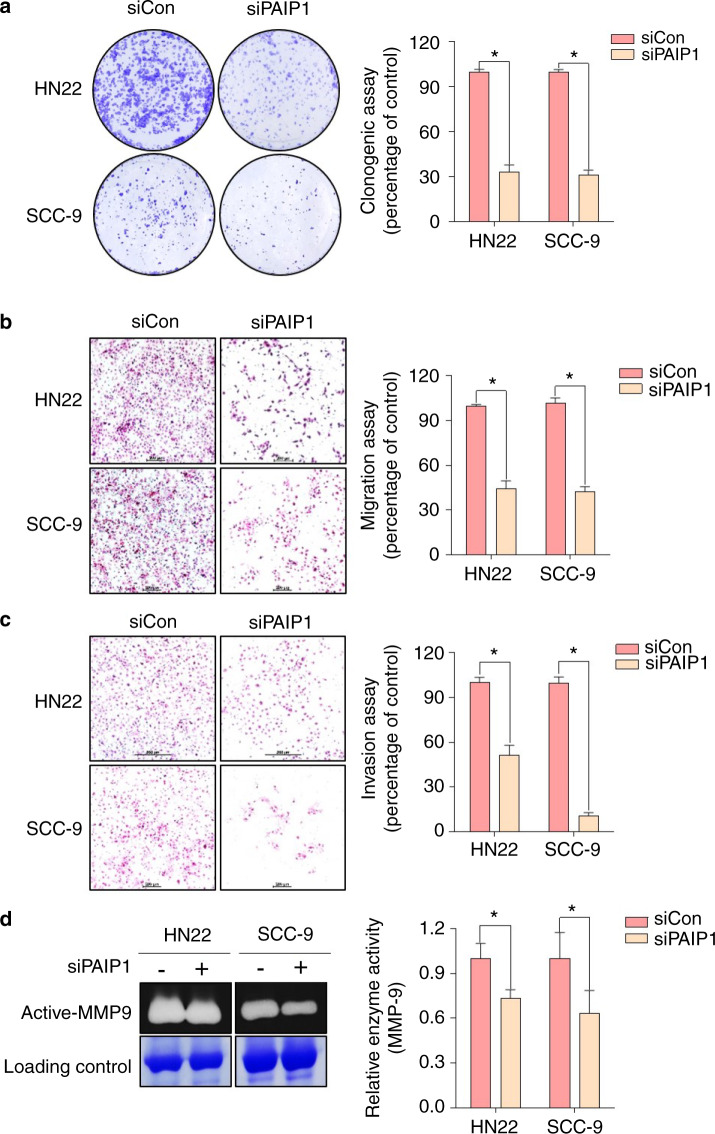
PAIP1 is essential for the invasiveness of OSCC cells. **a** Knockdown of PAIP1 reduced the colony forming abilities in OSCC cells. **b**, **c** PAIP1 inhibition decreased the migratory and invasive abilities of OSCC cells. **d** MMP9 activity in OSCC cells was examined using gelatin zymography. Data are represented as the mean ± SD of triplicates. *P < 0.05.

To identify whether PAIP1 is essential in OSCC progression, we knocked down PAIP1 in high PAIP1-expressing cell lines (HN22 and SCC-9) using siRNA interference (Fig. S5). First, we confirmed the effects of PAIP1 on colony forming abilities and found that PAIP1 downregulation was associated with a significant decrease in that ability (Fig. 4a). Moreover, knockdown of PAIP1 suppressed OSCC cell migration and invasion (Fig. 4b, c). To investigate how PAIP1 expression could affect the enzymatic activity of MMP9, we then performed gelatin zymography and found a significant decrease in MMP9 activity in both cell lines (Fig. 4d). These data indicated that PAIP1 could induce OSCC cell migration and invasion via upregulated MMP-9 activity.

### PAIP1 knockdown inhibited SRC phosphorylation in OSCC with metastasis

**Fig. 5:**
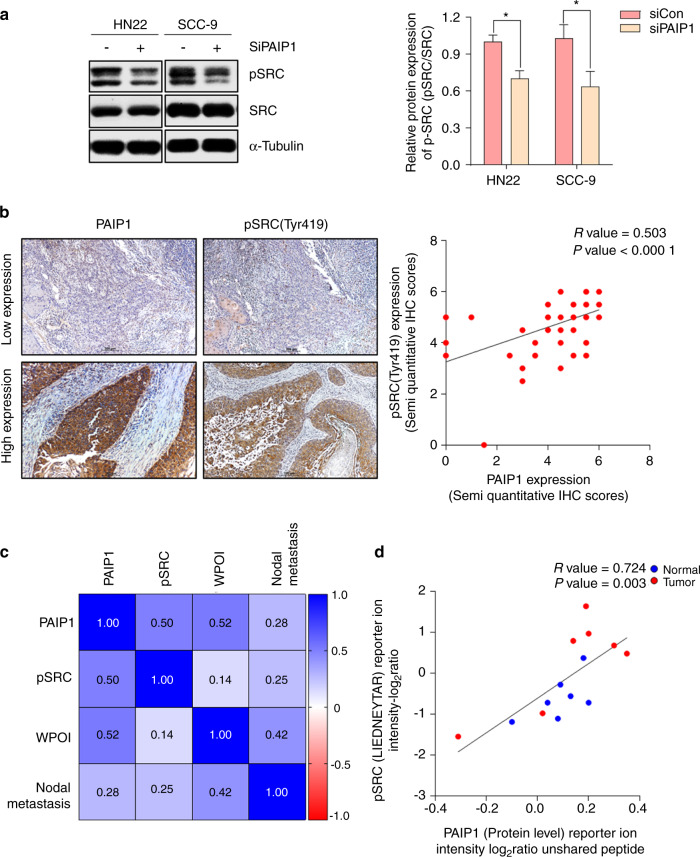
PAIP1 regulates the phosphorylation of SRC in OSCC with LNM. **a** PAIP1 inhibition significantly reduced the phosphorylation of SRC in OSCC cells. Data are represented as the means ± SD of triplicates. *P < 0.05. **b** Representative images show IHC staining of PAIP1 and pSRC (Tyr 419) in the same cases on serial sections for high and low expression groups and graphs show Pearson correlation in IHC scores of PAIP1 and pSRC expression levels. **c** Heat map demonstrates on positive multivariable Pearson correlation of PAIP1 and pSRC. **d** The graph show Pearson correlation in protein levels of PAIP1 and pSRC (LIEDNEyTAR) from CPTAC.

To examine the involvement of PAIP1 in SRC phosphorylation, closely related to tumor invasion and metastasis, we performed western blot in PAIP1 knockdown cells. The SRC (Tyr416) activation was strongly attenuated by PAIP1 inhibition in both cell lines (Fig. 5a). We then performed IHC for pSRC (Tyr 419) in serial sectioned OSCC cases stained for PAIP1 and found a significant Pearson correlation between PAIP1 and pSRC expression (Fig. 5b, *P*- and *R*-values of 0.000 1 and 0.50, respectively). We performed a multivariable analysis to evaluate the correlation between PAIP1, pSRC (Tyr 419), WPOI, and nodal metastasis, observing a positive correlation between these variables (Fig. 5c). Finally, as expected, the PAIP1 and pSRC correlation results were in good agreement with data accessed via proteomic data commons for PAIP1 and phosphorylated SRC peptide (LIEDNEyTAR) at the site tyrosine 419, for paired healthy and tumor samples (Fig. 5d, *P*- and *R*-values of 0.003 and 0.724, respectively). These data highlighted that SRC phosphorylation is involved in the poor OSCC prognosis with a high PAIP1 expression. A summary of the working model by which PAIP1 has metastatic potential and prognostic significance in OSCC is illustrated in Fig. S7.

## Discussion

PAIP1 has been identified as a target gene in 5p gain in cervical cancer^6^. Amplification of short arm of chromosome 5 is frequently seen in cancers^7,17,18^. This led us to hypothesize that PAIP1 might undergo amplification in head and neck cancer. Concurrent with our hypothesis, we found that the genetic alterations seen in head and neck cancers were mostly amplifications with mutations and fusions and a strong correlation between PAIP1 copy numbers and mRNA levels (Fig. S1a–c). Furthermore, we observed the correlation between the PAIP1 copy number and mRNA levels in the selected cohort from upper aerodigestive cancer cell lines and found a similar pattern (Fig. S1d). Therefore, these results suggest that a gain or amplification in the PAIP1 copy number levels is associated with a PAIP1 mRNA transcript level increase in cancer cells.

Recently, certain studies attempted to assess the correlation between PAIP1 expression and the prognostic value in different cancers. Indeed, we found a similar trend in the PAIP1 mRNA and protein level overexpression in oral cancers when compared to healthy controls (Fig. 1)^11–13,15,19^. Higher PAIP1 expression was a frequent event in cohorts of patients with OSCC, associated with higher risk and positive correlation with greater tumor size, LNM, and advanced stages of the tumor and reduced survival (Fig. 2 and Fig S3), consistent with that of various tumors^11–13,15,19^. Xie et al. described similar findings in tongue carcinomas, but the representative data does not corroborate with the conclusion^20^. Notably, PAIP1 expression in our OSCC samples appears to have a female predilection which has not yet been reported in other tumors (Fig. 2a, b). To investigate the said sexual predilection, the correlation was evaluated between the PAIP1 mRNA levels and AR ESR1, ERBB2, ESR2, PR, and the protein levels of Her2 and PR using the cBioportal and CPTAC data (Data not shown) but no significant correlation could be observed between any of the factors, similar to the findings in the case of breast cancer^11^. As far as we’re concerned that this is the first evidence demonstrating the correlation of a poor prognosis with PAIP1 expression in OSCC. These findings suggest that PAIP1 has appreciable potential and a possible clinical validity as a target for OSCC.

Tumor cells at the invasive fronts (most advanced layers or detached tumor cells) show molecular heterogeneity when compared to the preceding zones of the tumor, which can better predict the tumor outcomes^21–23^. In this study, we observed an interesting pattern of expression in which the levels of PAIP1 were higher in invading zones (ITF) when compared to inner regions (ITM) (Fig. 3a and Fig. S4a). PAIP1 expression was also associated with a malignant histopathological determinant, cellular cohesive, and non-cohesive invasion, which might clarify the aggressive behavior of cancers (Fig. S4b)^24,25^. The POI is a reportedly important predictor of LNM, indicating poor OSCC prognostic outcomes^26–28^. Based on our current results, WPOI has a positive correlation with LNM (Fig. 5c). Furthermore, advanced WPOI showed a strong correlation with high PAIP1 expression (Fig. 3b–d). However, PAIP1 expression did not significantly correlate with the histological differentiation of tumor (Figs. 3c, d and S4c). To the best of our knowledge, we described the first that higher PAIP1 was associated with WPOI, significantly greater in non-cohesive invasion patterns compared to cohesive invasion patterns. When all things considered, these results steered us to hypothesize that PAIP1 might play a pivotal role in OSCC with LNM.

Although the recently growing pieces of evidence suggest that high PAIP expression contributes to the aggressive behavior of various tumors, its role has never been determined in OSCC^11–13,15^. In particular, Guan et al. reported that PAIP1 knockdown in pancreatic cancer cells markedly decreased in the expression of MMP2, which affects the invasion and migratory process and MMP2 in OSCC has also been described as a potential biomarker for metastasis^15,29–33^, whereas we found no change in the MMP2 levels, rather MMP-9 activity downregulation in the PAIP1 knockdown of OSCC cells (Figs. 4d and S6)^15^. These results signify that PAIP1 may facilitate migration and invasion through differently expressed MMPs depending on cancer cell types. Besides MMPs expression, EMT-related molecules play an essential role in the cancer metastasis^34^. To analyze the influence of PAIP1 expression in the EMT process, we examined the expression of EMT-related molecules. In contrast to other reports, there was no significant relationship between PAIP1 and EMT-related molecules to regulate the metastatic cascade (Data not shown)^12,13,15^. Against to our expectations, these findings indicate that EMT-related molecules regulation of PAIP1 was unlikely to be pertinent in our model. If the migratory and invasive abilities of OSCC cells are blocked by PAIP1 knockdown, it may still occur by an alternative mechanism.

Several pathways induce cell motility and invasion thus influencing cancer malignant and suggesting an attractive potential therapeutic target for OSCC tumors^35,36^. Wang et al. reported that PAIP regulated AKT/GSK3β pathways for migration in lung cancer^14^. Although we did not find any significant changes including the AKT signaling pathway (data not shown), we for the first time found that PAIP1 was associated with activation of SRC by affecting phosphorylation of tyrosine residue 416/419 (Fig. 5). Following the identification of how PAIP1 affects SRC phosphorylation in the OSCC tissue samples, we found a positive correlation between the PAIP1 and pSRC levels. Furthermore, we observed the correlation associated with LNM using multivariable analysis (Fig. 5b–d). SRC is a proto-oncogene, which on activation, phosphorylation of TYR416/419 has pleiotropic roles^37^. SRC activity is generally regulated by phosphorylation and dephosphorylation of tyrosine domains or interaction with binding proteins like PDGFR, FAK which bind to the SH-2 domain^38^. This indicates that activated SRC can trigger numerous downstream targets to regulate carcinogenesis. Indeed, SRC activity has been described in HNSCC and implicated in tumor progression and metastasis^39,40^. Although the underlying mechanism of PAIP1 remains to be further elucidated in OSCC cells, these findings suggest that targeting of PAIP1 has a latent ability to inhibit OSCC metastasis by affecting SRC activity.

In conclusion, current findings suggest that PAIP1 expression increased the possibility of LNM and advanced tumor stage, additionally it was also positively associated with WPOI. We also found the correlation between PAIP1 expression and SRC phosphorylation in both in vitro and OSCC tissues. These results underline the potential of PAIP1 as a prognostic marker associated with LNM and a promising therapeutic target for patients with OSCC.

## Materials and methods

### In silico analysis

Three data repositories (TCGA, GEO, and *CPTAC*) were used to extract expression data and identify PAIP1 expression and its relationship with the clinicopathological features in HNSCC.

#### Gene Expression Omnibus (GEO Database)

First, PAIP1 mRNA expression was explored using the GEO database, https://www.ncbi.nlm.nih.gov/geo/. We performed the differential expression analysis of GSE30784^41^. We also, investigated the variations in PAIP1 mRNA levels between the normal cases, dysplasia cases, and cancer cases, using the geo series GSE30784 for the reporter id 213754_s_at and variation of the PAIP1 mRNA levels within the same cases between non-tumorous and tumorous epithelia, using the geo series GSE37991 for reporter id ILMN_1776398^42^. Furthermore, we also investigated the variation in the mRNA levels of PAIP1 between the negative and positive nodal metastasis, using the geo series GSE78060 for reporter id 213754_s_at. The gcRMA normalized values were extracted for different GSM samples within the same study set using the series matrix file. Extracted data were converted to a log2 scale for plotting and analysis. GEO2R was used to confirm normalization and analysis for all the extracted values^43^.

#### The Cancer Genome Atlas, Genomic Data Commons

HNSCC dataset was used to create a custom cohort, which was accessed via https://portal.gdc.cancer.gov/; it included lip, palate, gum, the base of the tongue, tonsil, the floor of the mouth, other and unspecified parts of the tongue, other and unspecified parts of the mouth, and other ill-defined sites in the lips, oral cavity, and pharynx. We analyzed the PAIP expression levels using the FPKM-UQ files. The files were downloaded manually, and the PAIP-related data was extracted using the LINUX system. The extracted data were converted to a log2 scale for further analysis. The extraction, sorting, and parsing code was custom-developed using Jupyter notebook and Pandas on top of Python 3.9, the code can be found at (https://github.com/kunalchawlaa/TCGA-Oral-Cancer). Reference for the codes developed was accessed from, https://labs.cd2h.org/gitforager/repository/repository.jsp?id=77901462. The results shown here are partially based upon data generated by the TCGA Research Network: https://www.cancer.gov/tcga.

#### Clinical Proteomic Tumor Analysis Consortium, Proteomic Data Commons

We analyzed the proteomic PAIP levels of a custom cohort, accessed from https://pdc.cancer.gov/pdc/ including the base of the tongue NOS (Not Otherwise Specified), cheek mucosa, lip NOS, gum NOS, tonsil NOS, the floor of the mouth NOS, head, face, or neck NOS, just as well as the overlapping lesions of the lips, oral cavity, and pharynx. The log values for reporter ion intensity for unshared PAIP1 peptides were extracted after the manual downloading of the files CPTAC3_Head_and_Neck_Carcinoma_Proteome.tmt11.tsv and CPTAC3_Head_and_Neck_Carcinoma_Phosphoproteome.phosphopeptide.tmt11.tsv. The obtained values were used to compare the physiological and cancer-affected expression levels^44^.

### Clinical samples

The specimens analyzed were retrieved from 58 OSCC patients who were surgically treated at the Seoul National University Dental Hospital at Department of Oral and Maxillofacial Surgery in 2009. The clinical data were collected from the patient medical records which included age, sex, recurrence, and survival of the cases. The tumor grading was based on the World Health Organization Classification of Tumors^45^ and staging of tumors was based according to the TNM system recommended by the American Joint Committee on Cancer^46^. We performed a histologic risk assessment to record the worst invasion patterns (WPOI)^26,27^. The clinicopathological and histological features of the patients with OSCC are summarized in Supplementary Tables 1 and 2. The Institutional Review Board (IRB) of Seoul National University Dental Hospital approved the research (IRB number: ERI20021).

### Immunohistochemistry

Four μm serial sections were obtained from 58 and 36 formalin-fixed and paraffin-embedded OSCC and adjacent healthy oral mucosal tissue specimens, respectively. The specimens were serially sectioned for each case to detect PAIP1 and pSRC (Tyr419). One section per case was immunohistochemically stained using a mouse monoclonal anti-PAIP1 antibody (1:300, Santa Cruz) after heat-induced antigen retrieval in a microwave oven for 10 min in citrate buffer (pH 6.0) incubated overnight at 4 °C, followed by detection using Dako real detect HRP-conjugated system (Dako). The other section was immunohistochemically stained after heat-induced antigen retrieval in a microwave oven for 8 min in citrate buffer (pH 6.0) using anti-pSRC (Tyr419) (1:250, Invitrogen, Thermo Fisher Scientific) incubated for 1 h at room temperature, followed by detection using Dako real detect HRP-conjugated system. Slides incubated without primary antibodies were used as a negative control.

### Immunohistochemical staining evaluation

Two oral pathologists (NS and SDH) assessed immunohistochemical staining semi-quantitatively, individually following blinding. In the case of disagreement, the scores were reached to a consensus following discussion. Tumor cells that demonstrated cytoplasmic staining were considered positive. The tumor was demarcated into two different zones: inner tumor mass and invasive tumor front (most advanced layers and detached tumor cells). Both zones were scored independently^21^ along with the total tumor score. The percentage of positive cells was scored as follows: *Score 0*, less than 5% positively stained cells; *Score 1*, 5%–25% positively stained cells; *Score 2*, 26%–50% positively stained cells; and *Score 3*, 51%–100% positively stained cells. The intensity was also scored for positive cells as follows: *Score 0*, negative; *Score 1*, weak (light brown); *Score 2*, moderate (brown); and *Score 3*, strong (dark brown). Each intensity score was added to the percentage score to produce a combined score. The final IHC score varies between 0 and 6. IHC scores between 0 and 2, less than 4, and equal to or greater than 4 were classified as negative, low, and high, respectively (Fig. S2). For statistical evaluation, the PAIP1 expression was dichotomized between the low (comprising negative and low cases) and high expression groups (comprising high cases), respectively.

### Cell culture

Eight OSCC cell lines were screened along with a positive and negative control for PAIP1 protein and mRNA expression. The Hokkaido University kindly provided the HSC2, HSC3, HSC4, Ca9.22, HO1-N1, and HO1-U1 cells and the Dankook University kindly provided the HN22 cells. The SCC-9 was acquired from the Korean Cell Line Bank and MDA-MB-231 cells were purchased from the American Tissue Culture Collection. The Yonsei University kindly provided the iHOK cells. The MDA-MB-231 and iHOK were used respectively as positive and negative controls. The cells were routinely maintained in DMEM-F12 or RPMI 1640 media, supplemented with 10% FBS and penicillin/streptomycin and KSF medium (for iHOK cells) supplemented with BPE/EGFR at 37 °C, 5% CO_2_ until reaching a confluency of 70%–80%.

### Quantitative RT-PCR

1 microgram of RNA was used to reverse transcribe using the AMPIGENE cDNA Synthesis Kit (Enzo Life Sciences, Inc.) according to the manufacturer’s protocol and then, the resultant cDNA was subjected to PCR using the AMPIGENE qPCR Green Mix Hi-Rox (Enzo Life Sciences, Inc.). The real-time PCR was performed using the Applied Biosystems StepOnePlus Real-Time PCR System (Applied Biosystems) and the amplification parameters for all genes were as follows: 95 °C for 2 min, followed by 40 cycles of 95 °C for 10 s, and 60 °C for 30 s. Fold amplification of each gene was normalized to the amount of GAPDH and calculated using the 2^−∆∆CT^ method^47^. We used the qPCR primers as follows: PAIP1 forward, 5′-TTT GGA AGA TGC TTG GAA GG-3′, PAIP1 reverse, 5′-TGA AGT TGC ATG GAC TCT GC-3′; GAPDH forward, 5′-GTGGTCTCCTCTGACTTCAAC-3′, GAPDH reverse, 5′-CCTGTTGCTGTAGCCAAATTC-3′.

### Immunoblotting

Protein lysates were prepared using RIPA buffer supplanted with a protease inhibitor cocktail. The concentration of protein was determined by the DC Protein Assay Kit. The equal amounts of protein samples were separated on SDS polyacrylamide gels and transferred to a polyvinylidene fluoride membrane. This membrane was blocked with a blocking solution, TBST, supplemented with 5% Difco Skim Milk (BD bioscience) for 1 h, the membrane was then incubated with PAIP1-specific antibody (Santa Cruz), anti-GAPDH antibody (Abcam), anti-α-Tubulin (Santa Cruz), anti-SRC (Cell Signaling Technology), anti-pSRC (Tyr416) (Cell Signaling Technology), or anti-MMP9 (Cell Signaling Technology) in TBST. Following incubation with HRP-conjugated secondary antibodies, proteins were finally detected using the SuperSignal West Pico Chemiluminescent Substrate and captured using ImageQuant LAS 500 (GE Healthcare Life Sciences).

### Small-interfering (siRNA) RNA transfection

Small-interfering RNAs (siRNA) targeting *PAIP1* were provided by Bioneer. The *PAIP1* siRNA sequences were as follows: human *PAIP1* siRNA (#10605-1): sense 5′-CCAGGUGGUUGUAGCUCCU-3′, antisense 5′-AGGAGCUACAACCACCUGG-3′. The cells were seeded onto 60 mm dishes. The cells were cultured along with 25 nmol·L^−1^ of sicontrol or PAIP1 siRNA using Lipofectamine 2000 reagent according to the manufacturer’s instructions.

### Clonogenic formation assay

*PAIP1* siRNA-treated cells were seeded onto six-well culture dishes (2000 cells per well) and incubated. The medium was changed every 3 days for a week. The colonies (clusters of 20 or more cells) were then stained with 1% crystal violet and taken under an inverted microscope, and the number of colonies was calculated using the Image J software.

### Transwell migration & invasion assays

The siRNA-treated cells (8 × 10^4^ cells per well) were grown in the upper chamber of transwell inserts in 500 μL of serum-free DMEM/F12 medium. To assess cell migration, 8 µm Transwell membranes of chamber (Corning) were precoated on the lower surface with Collagen Type I to yield a concentration of 0.5 mg·mL^−1^. For the invasion assays, the transwell inserts were precoated with growth factor reduced Matrigel (1:15 dilution). A total of 700 μL of DMEM-F12 with 10% FBS was inoculated in the lower chamber and then, the assay plates were incubated at 37 °C in 5% CO_2_ for 24 h. The upper chamber was then wiped off with a cotton swab, following this hematoxylin and eosin were used to stain the chamber. The cells were counted under a light microscope (Leica DM5000B; Leica Microsystems). Finally, 10 unique microscopic fields at ×100 magnification from each chamber were randomly chosen for cellular counting.

### Gelatin zymography

The siRNA-treated groups were sustained in a serum-free medium for 72 h. To collect samples of the MMP protein extract, the conditioned medium was concentrated using Amicon Ultra-15 Centrifugal Filter Units (Millipore) at 4500 g and 4 °C for 80 min. To assess the MMP-9 activities, 10 and 30 µg protein samples were applied to an acrylamide gel, 8% which was prepared with 1.5 mg·mL^−1^ gelatin. Following electrophoresis, renaturing of gel was performed using 2.5% Tween-20 solution at room temperature for 30 min. After renaturation, the gel was developed using zymogram incubation buffer (Novex, Thermo Fisher Scientific) at 37 °C for 72 h. The gel was then stained with Coomassie Brilliant Blue R250 (Bio-Rad Laboratories). Thereafter, de-staining of the gel was performed using a solution of 40% methanol and 10% acetic acid, until the MMP-9-degraded part of the membrane became clear. Evaluation of the density of the clear bands was done using the ImageJ software.

### Statistical analysis

Statistical tests were conducted using the SPSS ver. 25.0 (SPSS, Inc.) and GraphPad Prism 8 software. All results were expressed as the means ± standard deviation (SD). Multivariable correlations between the immunohistochemical staining and clinicopathological parameters of the tumors were performed via multivariable Pearson correlation, Student’s t-test, the Chi-square test, and Fisher’s exact test. The relative risks were calculated for different variables for high PAIP1 expressions. The statistical significance of the in vitro experimental data was analyzed using the two-tailed Student’s t-test (for comparisons of two treatment groups) or one-way ANOVA with Tukey’s post hoc test (for comparisons of three or more groups). A *P* < 0.05 was considered statistically significant.

## Supplementary information

Supplementary Figure Legends

Figure S1

Figure S2

Figure S3

Figure S4

Figure S5

Figure S6

Figure S7

Supplementary Tables

## Data Availability

All publicly accessed data are available on databases (TCGA, GEO, CCLE, and CPTAC) described in methodology. Analyzed datasets used during the study can be made available from the corresponding author upon reasonable request.
