# Supplementary material for: Effect of PAIP1 on the metastatic potential and prognostic significance in oral squamous cell carcinoma
Source: Int J Oral Sci. 2022 Feb 14;14:9. doi: 10.1038/s41368-022-00162-8 (PMC8841500; doi:10.1038/s41368-022-00162-8)
Supplement: Supplementary file 1 — Supplementary Figure Legends [file 41368_2022_162_MOESM1_ESM.docx]

**Supplementary Figure Legends**

**Fig. S1** PAIP1 is up-regulated across different cancers and HNSCC. **a** copy number alterations levels of PAIP1demonstrated frequent amplification across various cancers, using cBioportal. **b** PAIP1 mRNA levels were up-regulated across various cancers like Lung, Head and Neck, Esophageal, etc. **c** Positive correlation of PAIP1 mRNA expression levels versus PAIP1 copy number values in head and neck cancers, TCGA data acquired using cBioportal, r value 0.81 *p* value 0.000. **d** Positive correlation of PAIP1 mRNA expression levels versus PAIP1 copy number values in various oral cancer cell lines using CCLE, r value 0.707 *p* value 0.000.

**Fig. S2** PAIP1 expression patterns in OSCC. PAIP1 IHC scores were classified as Negative, Low expression, High expression.

**Fig. S3** PAIP1 expression is significantly correlated with poor survival of HNSCC patients. Kaplan Meier analysis demonstrated that high PAIP1 expression was associated with decreased overall survival, plot generated using KM plotter [<http://kmplot.com/analysis/>] for HNSCC cohort.

**Fig. S4** Immunohistochemical scores for histopathological evaluation. **a** Graph represented IHC scores of PAIP1 in ITM (inner tumor mass) and ITF (invasive tumor front). The statistical comparisons between groups were evaluated using a paired t-test. **b** Graph represented IHC scores of PAIP1 in cohesive and non-cohesive pattern of invasion. The statistical comparisons between groups were evaluated using student t-test. **c** PAIP1 proteomic expression did not significantly correlate with histological differentiation of OSCC in CPTAC custom cohort. The statistical comparisons between groups were evaluated using student t-test.

**Fig. S5.** Low concentration of PAIP-1 siRNA inhibits the expression of PAIP1 protein without cell viability in OSCC cells. **a-b** PAIP1 expression in HN22 and SCC-9 cells transfected with PAIP1 siRNA or control siRNA was assessed by Western blot analysis. **c** Cell viability was determined using a trypan blue exclusion assay. Data are expressed as means ± standard deviation (SD) of triplicate experiments. Compared with control group: *p < 0.05.

**Fig. S6** Effect of PAIP1 on cellular MMP9 levels. Knockdown of PAIP1 significantly reduced cellular levels of MMP9 in HN22 cells, but no effect in SCC9 cells. Graphs show the mean ± SD of triplicate experiments and significance compared with the control (* p < 0.05)

**Fig. S7** A proposed working model of the role of PAIP1 in oral squamous cell carcinoma. PAIP1 is related with lymph node metastasis and invasion by SRC and MMP9.

**Supplementary Methods**

*cBioportal(supple)*

To analyze genetic alterations associated with PAIP1 across epithelial origin tumors of different organs, a pan cancer analysis was done for TCGA PanCan 2018 datasets for various tumors, using cBioportal (<https://www.cbioportal.org/>). Datasets for Clear Cell Renal Cell Carcinoma and Papillary Renal Cell Carcinoma were combined into single entry of Renal cancers and datasets for Glioblastoma Multiforme and Low-Grade Glioma were combined into single entry of Brain cancers. Tumors of mesenchymal origin and tumors with hybrid nature were not included for analysis and evaluation. Followed by analysis of genetic alterations, mRNA levels of PAIP1 were assessed for Head and Neck Cancer database in relation to log2 of copy-number values of PAIP1 [1].

*UALCAN*

Firstly, a pan cancer analysis was done across TCGA Pan Cancer datasets for various tumors, using UALCAN, http://ualcan.path.uab.edu/index.html, to analyze variation in mRNA expression levels of PAIP1 across epithelial origin tumors of different organs. The distribution values were recorded and replotted [2].

*Cancer Cell Line Encyclopedia (CCLE)*

Following the assessment of clinical databases, cell line database, <https://portals.broadinstitute.org/ccle> was screened. A custom cohort was made from upper aerodigestive cell lines, including cell lines from tongue squamous cell carcinoma, squamous cell carcinoma of lower alveolus, oral cavity squamous cell carcinoma, gingival squamous cell carcinoma, head and neck basaloid carcinoma; to evaluate PAIP1 mRNA level in relation to PAIP1 copy number values.

*Kaplan-Meier Plotter*

Online available software, <http://kmplot.com/analysis/index.php?p=background>, was used to analyze and plot the association of PAIP1 levels to survival in HNSCC patients. The software uses TCGA, GEO and EGA database for the mRNA values [3].

*Trypan blue exclusion assay*

HN22 and SCC-9 cells were treated with different concentration of PAIP1 siRNA for 24 h, and cell viability was measured using trypan blue staining (Gibco, paisley, UK). Cells were stained with 0.4% trypan ble solution, and viable cells were counted using a hemocytometer.

**Supplementary References**

1. Cerami E, Gao J, Dogrusoz U, Gross BE, Sumer SO, Aksoy BA *et al*: The cBio cancer genomics portal: an open platform for exploring multidimensional cancer genomics data. Cancer Discov. 2012;2(5):401-404.

2. Chandrashekar DS, Bashel B, Balasubramanya SAH, Creighton CJ, Ponce-Rodriguez I, Chakravarthi B *et al*: UALCAN: A Portal for Facilitating Tumor Subgroup Gene Expression and Survival Analyses. Neoplasia. 2017;19(8):649-658.

3. Nagy Á, Munkácsy G, Győrffy B: Pancancer survival analysis of cancer hallmark genes. bioRxiv. 2020:2020.2011.2013.381442.
