# Supplementary figures and images for: Effect of PAIP1 on the metastatic potential and prognostic significance in oral squamous cell carcinoma
Source: Int J Oral Sci. 2022 Feb 14;14:9. doi: 10.1038/s41368-022-00162-8 (PMC8841500; doi:10.1038/s41368-022-00162-8)

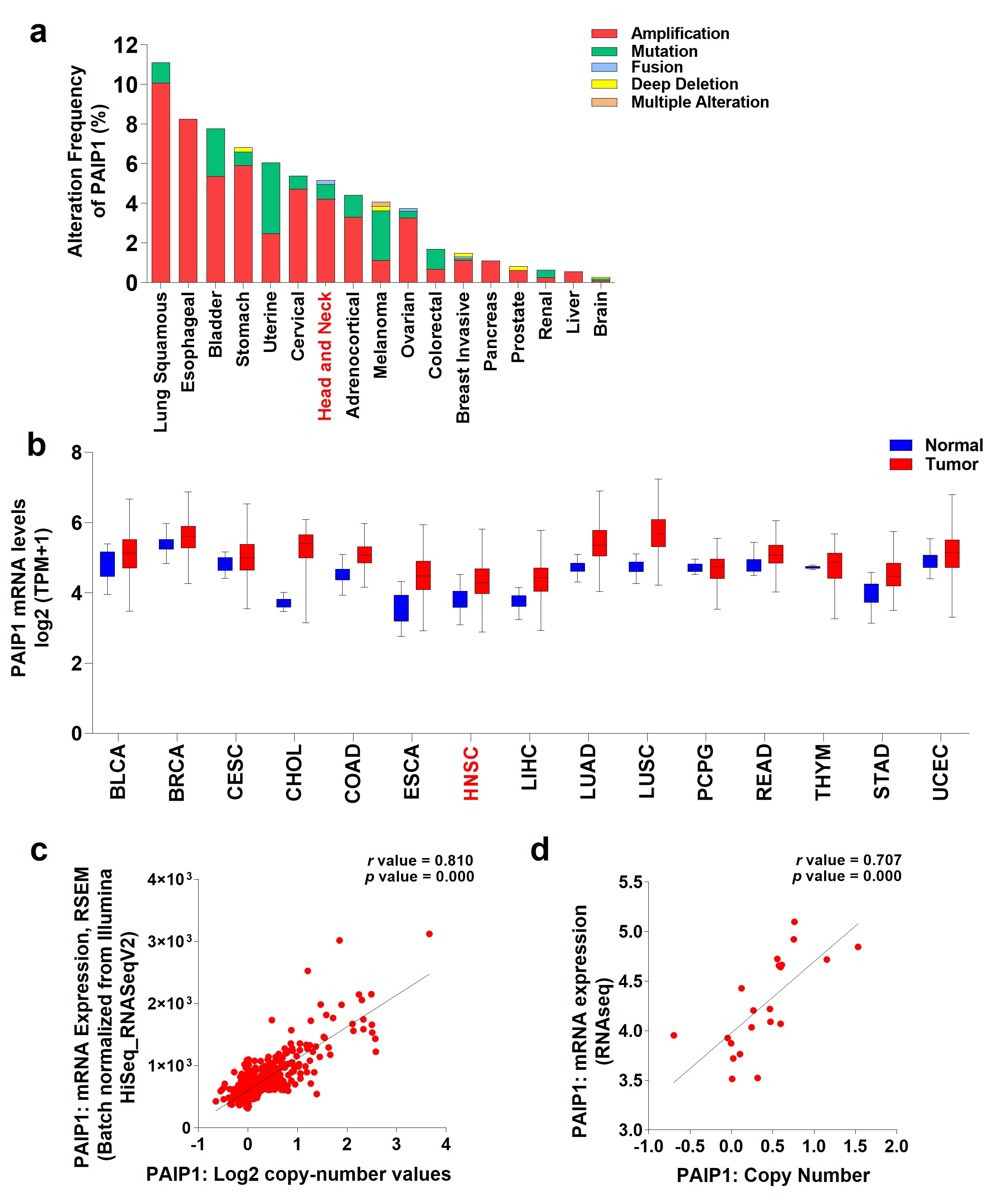

Supplement: Supplementary file 2 — Figure S1 [file 41368_2022_162_MOESM2_ESM.jpg]

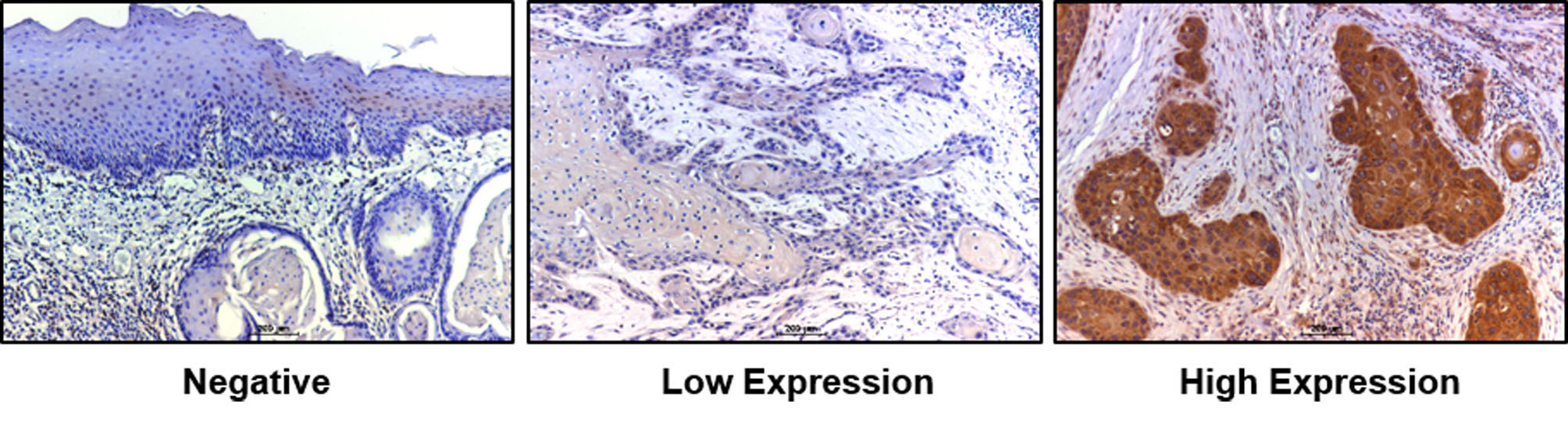

Supplement: Supplementary file 3 — Figure S2 [file 41368_2022_162_MOESM3_ESM.jpg]

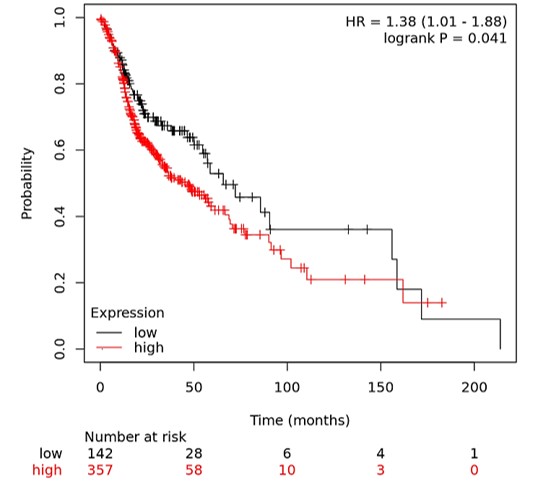

Supplement: Supplementary file 4 — Figure S3 [file 41368_2022_162_MOESM4_ESM.jpg]

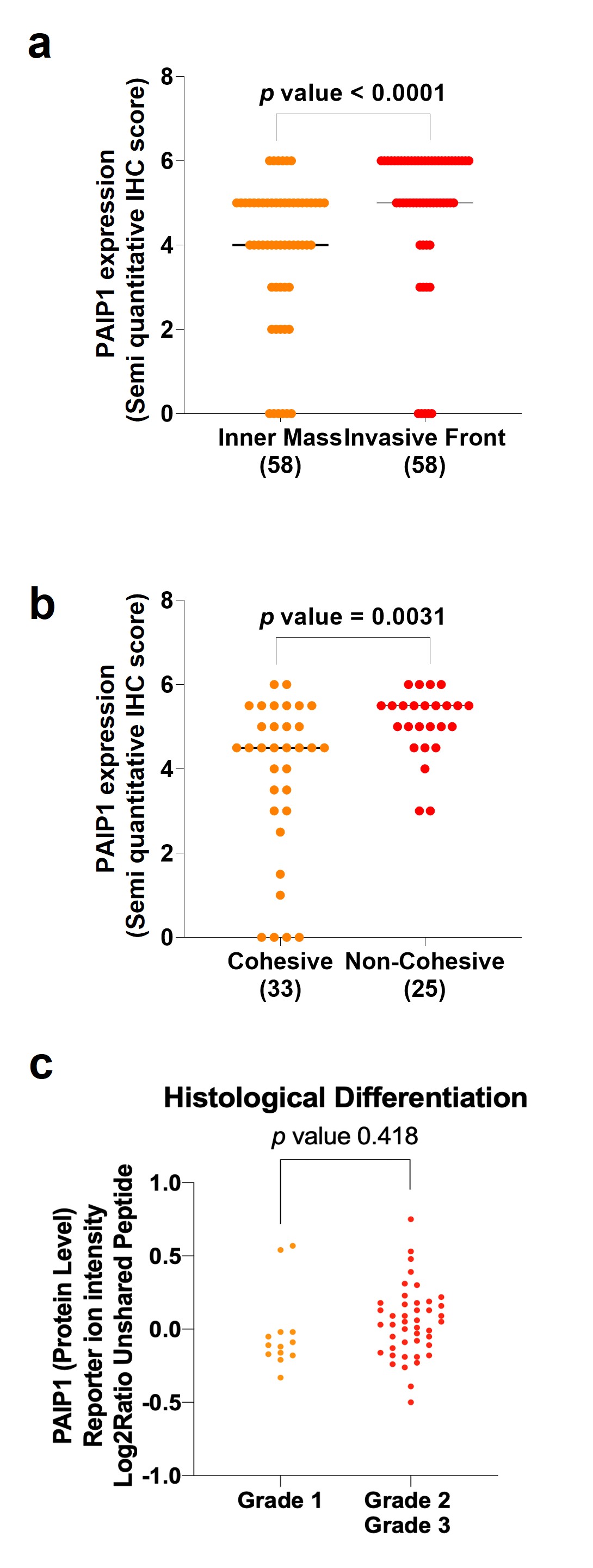

Supplement: Supplementary file 5 — Figure S4 [file 41368_2022_162_MOESM5_ESM.jpg]

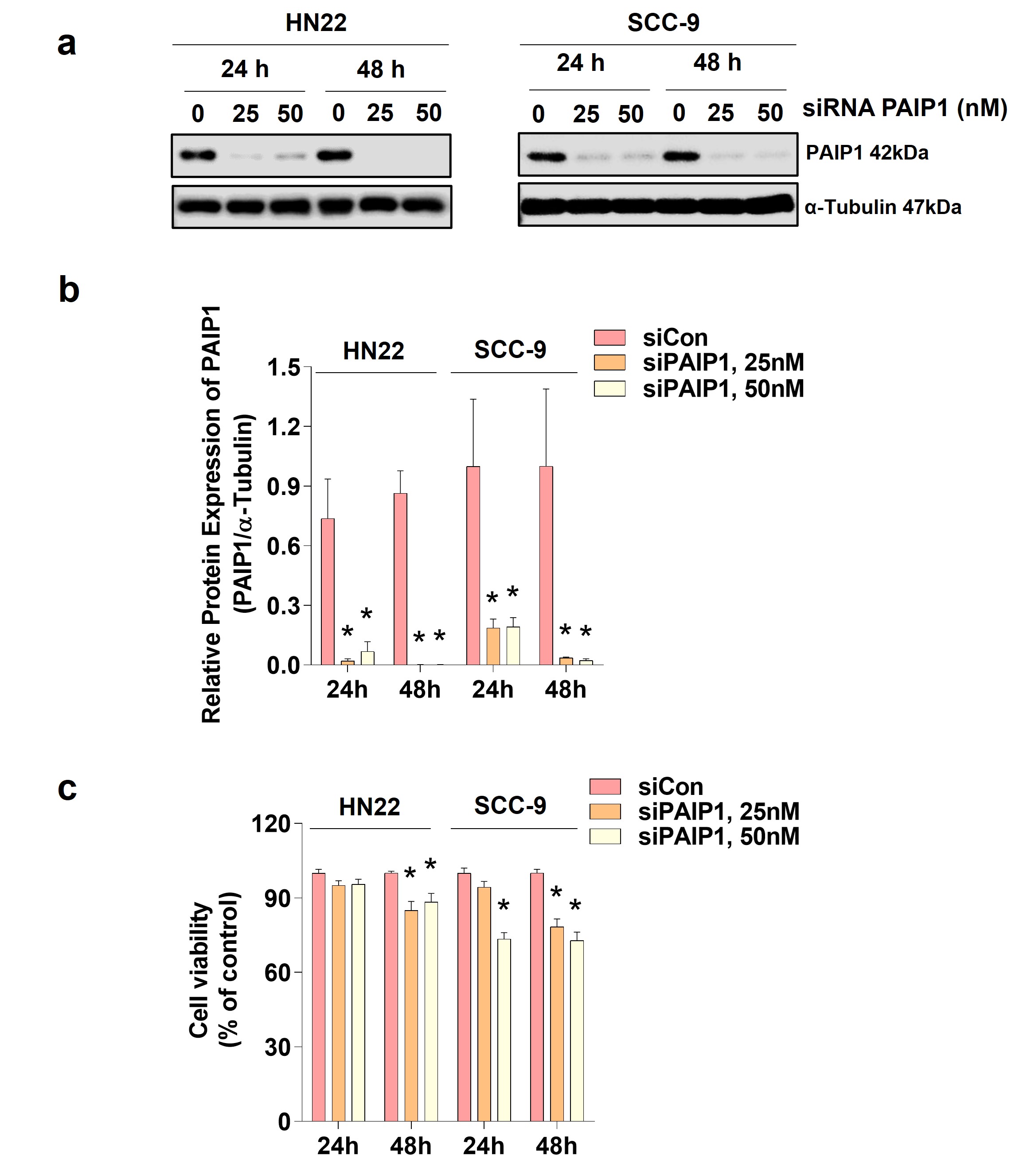

Supplement: Supplementary file 6 — Figure S5 [file 41368_2022_162_MOESM6_ESM.jpg]

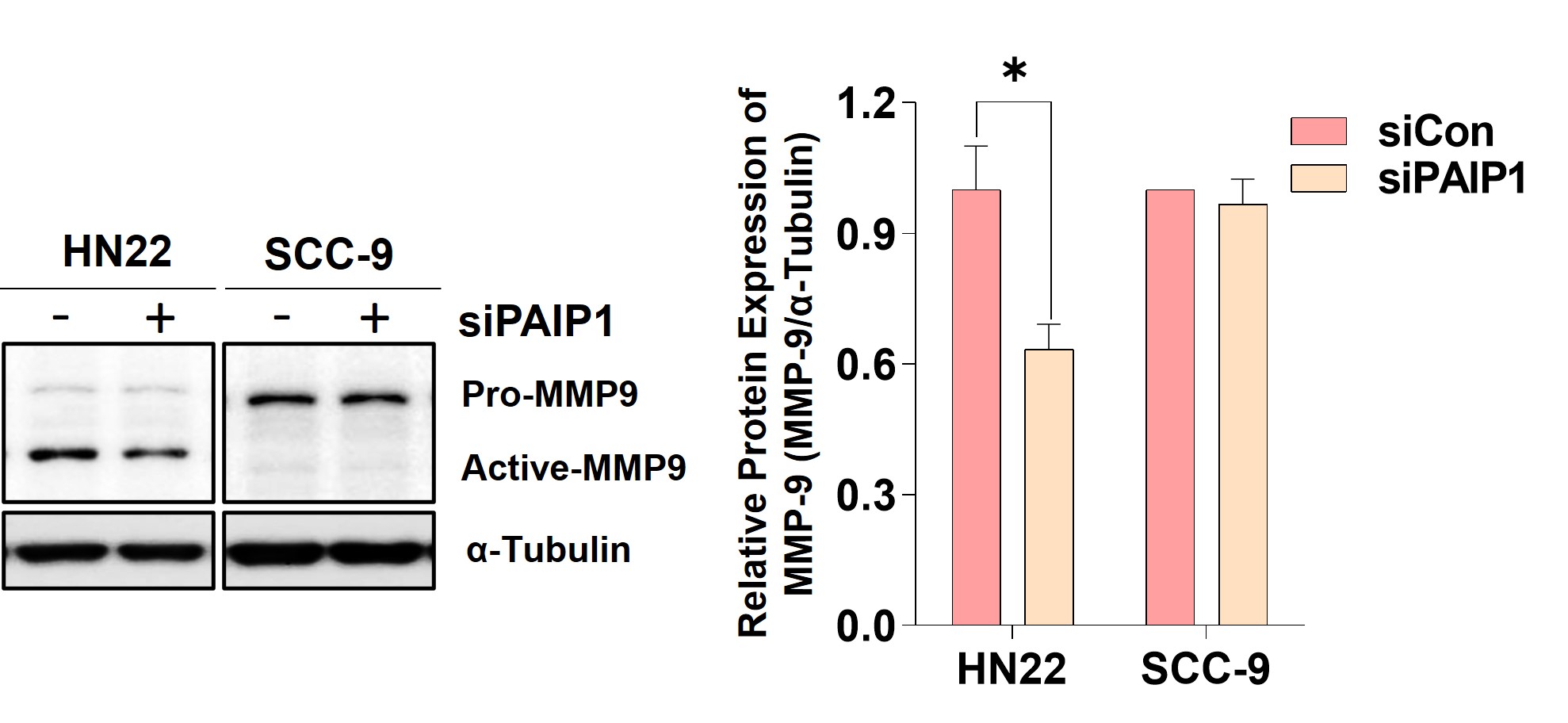

Supplement: Supplementary file 7 — Figure S6 [file 41368_2022_162_MOESM7_ESM.jpg]

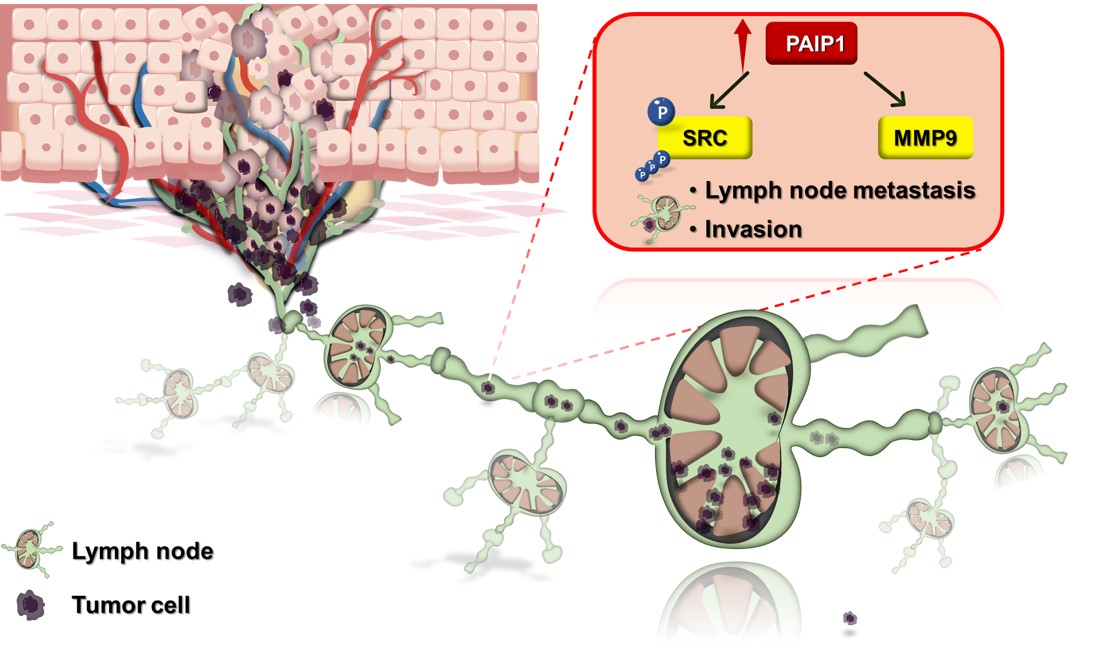

Supplement: Supplementary file 8 — Figure S7 [file 41368_2022_162_MOESM8_ESM.jpg]
