# Supplementary material for: Effect of PAIP1 on the metastatic potential and prognostic significance in oral squamous cell carcinoma
Source: Int J Oral Sci. 2022 Feb 14;14:9. doi: 10.1038/s41368-022-00162-8 (PMC8841500; doi:10.1038/s41368-022-00162-8)
Supplement: Supplementary file 9 — Supplementary Tables [file 41368_2022_162_MOESM9_ESM.docx]

**Supplementary Table 1. Clinical Characteristics of OSCC Patients (58 cases)**

| **Variable** | **No. cases 58 (%)** |
| --- | --- |
| Age (y); mean, 62.9 |  |
| 30-39 | 1 (1.72) |
| 40-49 | 4 (6.90) |
| 50-59 | 17 (29.31) |
| 60-69 | 17 (29.31) |
| 70-79 | 18 (31.03) |
| 80-89 | 1 (1.72) |
|  |  |
| Sex |  |
| Male | 34 (58.62) |
| Female | 24 (41.38) |
|  |  |
| T classification |  |
| T1 | 11 (18.97) |
| T2 | 16 (27.59) |
| T3 | 8 (13.79) |
| T4a | 22 (37.93) |
| T4b | 1 (1.72) |
|  |  |
| N classification |  |
| N0 | 40 (68.97) |
| N1 | 2 (3.45) |
| N2b | 10 (17.24) |
| N2c | 6 (10.34) |
|  |  |
| M classification |  |
| M0 | 56 (96.55) |
| M1 | 2 (3.45) |
|  |  |
| Stage |  |
| I | 11 (18.97) |
| II | 12 (20.69) |
| III | 3 (5.17) |
| IV | 31 (53.45) |
| IVb | 1 (1.72) |
|  |  |
| Recurrence |  |
| No | 49 (84.48) |
| Yes | 9 (15.52) |

**Supplementary Table 2. Histological Variables Worst Pattern of Invasion (WPOI) and grade**

| **Variable** | **No. cases 58 (%)** |
| --- | --- |
| WPOI |  |
| Type 1 | 3 (5.17) |
| Type 2 | 4 (6.90) |
| Type 3 | 26 (44.83) |
| Type 4 | 16 (27.59) |
| Type 5 | 9 (15.52) |
| Differentiation status |  |
| Well | 52 (89.66) |
| Moderately | 3 (5.17) |
| Poorly | 3 (5.17) |

**Supplementary Table 3. Association of PAIP1 with clinicopathological features of oral squamous cell carcinoma**

| **Variable** | **No. cases**  **(n = 58)** | | **PAIP1** | | **Risk Ratio**  **(95% CI)** | ***p*** |
| --- | --- | --- | --- | --- | --- | --- |
|  |  |  | **Low Expression**  **(n = 13)** | **High Expression**  **(n=45)** |  |  |
| Age (y) | |  |  |  |  | 0.29 |
| <63 | | 28 | 8 | 20 | 0.86(0.65,1.14) |  |
| ≥63 | | 30 | 5 | 25 | 1.17(0.88,1.55) |  |
|  | |  |  |  |  |  |
| Sex | |  |  |  |  | **0.02** |
| Male | | 34 | 11 | 23 | 0.74(0.57,0.96) |  |
| Female | | 24 | 2 | 22 | 1.36(1.04,1.76) |  |
|  | |  |  |  |  |  |
| Tumor size | |  |  |  |  | **0.02** |
| T1 + T2 | | 27 | 10 | 17 | 0.7(0.51,0.95) |  |
| T3 + T4 | | 31 | 3 | 28 | 1.43(1.05,1.96) |  |
|  | |  |  |  |  |  |
| Lymph node metastasis | |  |  |  |  | **0.01** |
| Negative | | 40 | 12 | 28 | 0.74(0.59,0.93) |  |
| Positive | | 18 | 1 | 17 | 1.35(1.07,1.7) |  |
|  | |  |  |  |  |  |
| Distant metastasis | |  |  |  |  | 0.53 |
| Negative | | 56 | 12 | 44 | 1.57(0.39,6.33) |  |
| Positive | | 2 | 1 | 1 | 0.64(0.16,2.56) |  |
|  | |  |  |  |  |  |
| Stage | |  |  |  |  | **0.01** |
| I + II | | 23 | 10 | 13 | 0.62(0.43,0.9) |  |
| III + IV | | 35 | 3 | 32 | 1.52(1.11,2.35) |  |
|  | |  |  |  |  |  |
| Recurrence | |  |  |  |  | 0.26 |
| No | | 49 | 12 | 37 | 0.85(0.64,1.12) |  |
| Yes | | 9 | 1 | 8 | 1.18(0.89,1.56) |  |

**Supplementary Table 4. Association of PAIP1 with histopathological features of oral squamous cell carcinoma**

| **Variable** | **No. of cases**  **(n = 58)** | **PAIP1** | | **Risk Ratio**  **(95% CI)** | ***p*** |
| --- | --- | --- | --- | --- | --- |
|  |  | **Low expression**  **(n = 13)** | **High expression**  **(n=45)** |  |  |
| WPOI |  |  |  |  | **0.02** |
| Cohesive (I + II + III) | 33 | 11 | 22 | 0.72(0.55,0.95) |  |
| Non-Cohesive (IV + V) | 25 | 2 | 23 | 1.38(1.06,1.8) |  |
| Differentiation status |  |  |  |  | 0.69 |
| Well | 52 | 12 | 40 | 0.92(0.63,1.36) |  |
| Moderately to poorly | 6 | 1 | 5 | 1.08(0.74,1.6) |  |
